# Efficacy and safety of tenofovir in a kidney transplant patient with chronic hepatitis B and nucleos(t)ide multidrug resistance: a case report

**DOI:** 10.1186/1752-1947-8-281

**Published:** 2014-08-21

**Authors:** Chun Shan, Guo Qing Yin, Pei Wu

**Affiliations:** 1 Department of Infectious Disease, Nanjing Zhong-Da Hospital, Southeast University School of Medicine, 87 Ding Jia Qiao, Nanjing 210009, Jiangsu, China

**Keywords:** Hepatitis B, Kidney transplantation, Multidrug resistance, Nucleos(t)ide analogs, Tenofovir

## Abstract

**Introduction:**

Five nucleos(t)ide analogs are used to treat chronic hepatitis B. Ideal nucleos(t)ide analog therapy in chronic hepatitis B patients with kidney transplantation must ensure virological suppression and minimize renal injury. However, resistance to nucleos(t)ide analogs frequently results in virological breakthrough, hepatitis flare, and complicated deterioration of the transplanted kidney. Inappropriate rescue therapy for drug resistance may subsequently cause hepatitis B virus multidrug resistance. Currently, tenofovir is used to treat chronic hepatitis B patients with kidney transplantation. In the field, we first reported combination therapy with tenofovir plus entecavir in a kidney transplant chronic hepatitis B patient with nucleos(t)ide analog multidrug resistance.

**Case presentation:**

A 50-year-old Chinese man with chronic hepatitis B and kidney transplantation received nucleos(t)ide analog therapy with sequential monotherapy and combination therapy. Virological parameters, hepatic enzymology and renal function were monitored. Drug-resistance mutations were detected by sequence analysis. Our patient received sequential nucleos(t)ide analog monotherapy and inappropriate combination therapy during 132 months, which caused multidrug resistance and renal functional injury. Entecavir plus adefovir was administered in month 106, resulting in decreased hepatitis B virus load, normal hepatic function, and stabilized creatinine clearance. As a result of rebounded viral load and significantly declining creatinine clearance, tenofovir plus entecavir was administered in month 133. After eight weeks, undetectable hepatitis B virus DNA, normal hepatic function and improved creatinine clearance were present. Compared with combination therapy with adefovir plus entecavir, tenofovir plus entecavir showed a potent antiviral effect for multidrug resistance and minimized renal injury.

**Conclusions:**

In chronic hepatitis B patients with kidney transplantation, sequential monotherapy with antiviral agents with low barriers to resistance should be avoided, and initial therapy with entecavir is a better option. Combination therapy with tenofovir plus entecavir in this setting with multidrug resistance is safe and effective.

## Introduction

Approximately 350 to 400 million people worldwide are chronically infected with hepatitis B virus (HBV) [1,2]. As a result of viral replication and the host immune response, these patients may develop progressive chronic hepatitis B (CHB). Drugs for treating CHB include interferon (IFN) and nucleos(t)ide analogs (NAs). The advantages of IFN are the absence of resistance and the potential for immune-mediated control of HBV infection, but its disadvantages include severe side effects, contraindication in female patients during pregnancy, and aggravating illness in patients with decompensated cirrhosis or autoimmune disease. In particular, IFN should be avoided in patients with organ transplantation because of the risk of acute rejection [1,3]. Therefore, CHB patients with kidney transplantation should simultaneously receive immunosuppressive agents and NAs, instead of IFN.

NAs can be classified into nucleosides (lamivudine, telbivudine, emtricitabine and entecavir) and nucleotides (adefovir and tenofovir). Five NAs, namely lamivudine, adefovir, entecavir, telbivudine and tenofovir, are currently approved worldwide for CHB treatment [1,2]. In China, lamivudine was the only available antiviral drug for HBV infection before 2005, adefovir, entecavir and telbivudine were approved for CHB treatment between 2005 and 2007 [4], and tenofovir was used to treat HBV resistance in 2012. As a result of cost constraints, prescription regulations, or both, a considerable proportion of physicians in China still start monotherapy with NAs that are low barriers to resistance, including lamivudine, adefovir and telbivudine. These inadequate therapies may induce HBV resistance, or even multidrug resistance. At present, combination therapy is recommended for CHB with resistant mutations. An ideal combination therapy should include drugs with different mechanisms of action, such as a combination of entecavir plus adefovir or entecavir plus tenofovir [2].

Kidney transplantation is the best treatment for end-stage kidney disease. HBV infection in kidney transplant recipients still constitutes a major problem because CHB flare is a significant cause of mortality after transplantation. Compared with IFN, NAs are currently more efficient and safe for treating CHB after kidney transplantation. However, multiple factors limit NA administration, including the risk of kidney injury, the low barrier to resistance, and cross-resistance mutation in different NAs [2,5,6]. Infectious disease physicians do not understand fully the basic principles of resistance or management strategies of kidney injury in this setting, therefore, inadequate NA therapy can increase the risk of kidney injury, develop multidrug resistance, and even result in progressive liver disease.

Here we present a CHB patient with kidney transplantation who developed viral breakthrough, multidrug resistance, and complicated renal function decline during NA treatment. Finally, combination therapy with tenofovir plus entecavir resulted in undetectable HBV load and improvement of renal function.

## Case presentation

**Figure 1:**
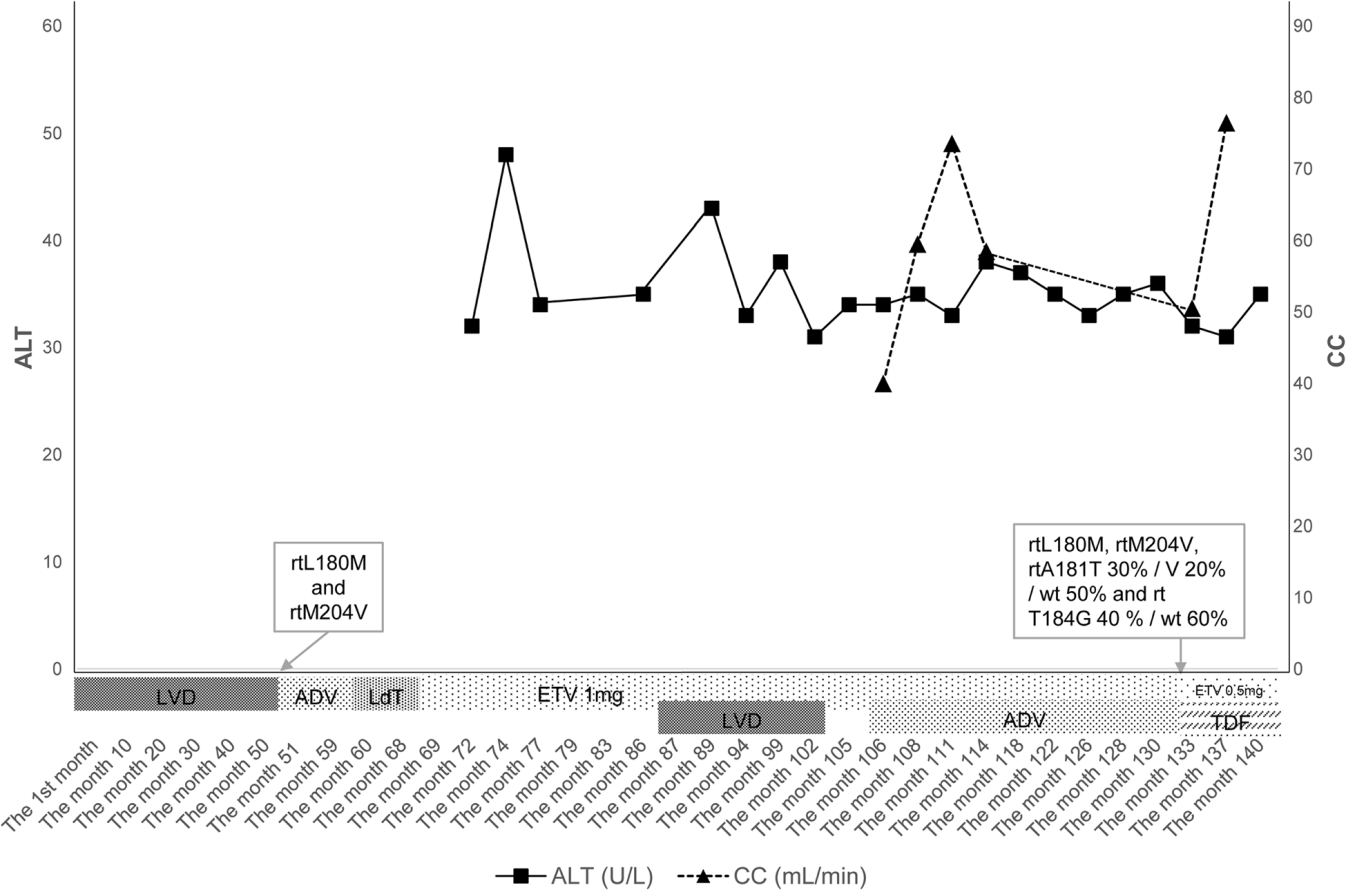
**Alanine aminotransferase and creatinine clearance levels during nucleos(t)ide analog therapy.** Because various physicians in various hospitals have separately participated in nucleos(t)ide analog therapy in different phases, parameters of liver enzymes, renal function and DNA load from month 1 to month 60 have not been recorded. Fluctuating alanine amino transferase levels showed during nucleos(t)ide analog therapy. Two bottom of creatinine clearance were present in month 104 and in month 133. The box showed the nucleos(t)ide analog-resistant mutations by sequence analysis. LVD, lamivudine; ETV, entecavir; ADV, adefovir; LdT, telbivudine; TDF, tenofovir; wt,wild-type.

**Figure 2:**
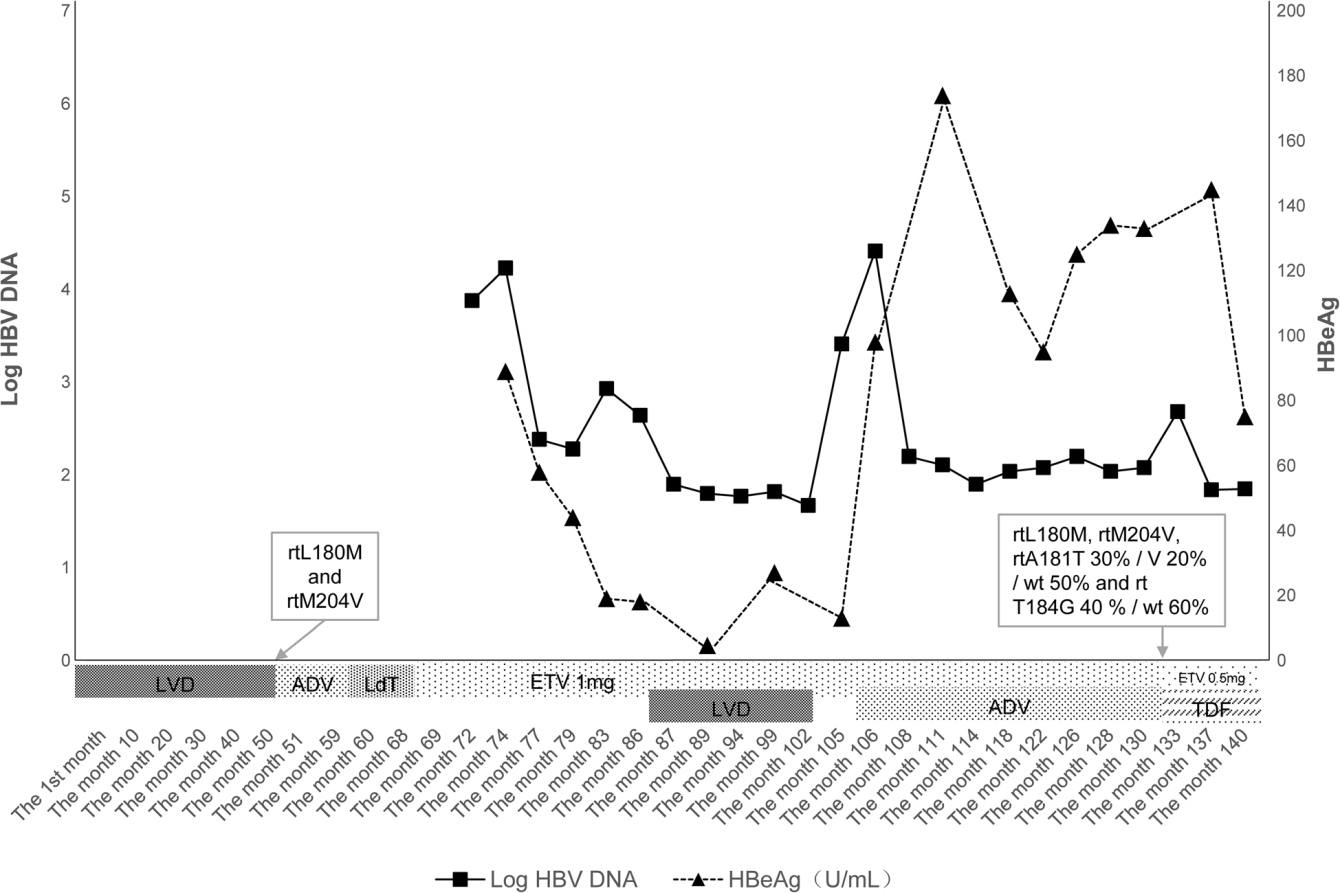
**Hepatitis B virus DNA and hepatitis B e antigen levels during nucleos(t)ide analog therapy.** Viral load rebound presented in month 104 and in month 133. Combination therapy with entecavir 1mg plus adefovir 10mg daily was applied to inhibit viral load in month 106. Multidrug resistance mutations, namely rtL180M, rtM204V, rtA181T 30%/V 20%/wt 50% and rt T184G 40%/wt 60%, were identified in month 133. Thus, combination treatment with entecavir 0.5mg plus tenofovir 300mg daily was immediately selected in this time point. Viral load gradually decreased, and creatinine clearance correspondingly increased.

A 50-year-old Chinese man underwent kidney transplantation for chronic renal failure and complicated CHB. He suffered glomerulonephritis complicating renal failure without detectable HBV markers and underwent hemodialysis for two years. Finally, kidney transplantation was performed. Immunosuppression and anti-rejection therapy, namely mycophenolatemofetil, tacrolimus and prednisolone, were routinely administered after kidney transplantation. As a result of the side effects of tacrolimus, he developed the postoperative complication of type 2 diabetes mellitus, and insulin was administered to control hyperglycemia. On hemodialysis, he was positive for hepatitis B surface antigen, hepatitis B e antigen (HBeAg), hepatitis B core antibody and HBV DNA (CobasAmplicor, Roche Diagnostics, Basel, Switzerland; low limit of quantification: 60IU/ml) with normal liver enzymes and was diagnosed with asymptomatic HBV infection. At age 39 years, he had active hepatitis B with elevated alanine aminotransferase (ALT) and aspartate aminotransferase (AST) but normal creatinine clearance (CC). Monotherapy with lamivudine 100mg daily was immediately administered, and this time point was identified as month 1 (Figure 1). Before 2005, monotherapy with lamivudine was the only choice. After 51 months, virological breakthrough and hepatitis flare were detected, and lamivudine resistance was detected by sequence analysis (Applied Biosystems 3500 Series Genetic Analyzer; Life Technologies, Carlsbad, CA, USA). A series of monotherapies was performed, namely adefovir 10mg daily for eight months, telbivudine 600mg daily for eight months, and entecavir 1.0mg daily for 17 months. Unfortunately, increased HBV load (10^3^ to 10^4^IU/ml), positive HBeAg and fluctuating ALT values within three times the upper limit f normal (ULN) were shown during the three years. Sequential monotherapy resulted in virological breakthrough, resistance mutation, and partial virological response, hence combination therapies were chosen. An inappropriate regimen, lamivudine 100mg plus entecavir 1mg daily, was administered in month 87, in an attempt to inhibit HBV and avoid continuous renal damage. Following this therapy, viral load decreased to 100IU/ml after two months, but rebounded to 2.6×10^3^IU/ml in month 104. Resistance mutations with rtL180M and rtM204V were again detectable. Renal function showed that serum creatinine levels were at ULN, and CC was 59.5ml/min. Tenofovir had not yet been approved in China by 2011.Therefore alternative therapy with entecavir 1mg plus adefovir 10mg daily was applied to inhibit viral load in month 106. Three months later, this treatment decreased viral load to 130IU/ml, and maintained stable CC, fluctuating between 59.5 and 73.6ml/min. In month 114, viral load was 80IU/ml (low limit of quantification: 20IU/ml), and CC 58.5ml/min. However, in month 133, viral load rebounded to 480IU/ml, CC decreased to 50.5ml/min, and multidrug resistance mutations, namely rtL180M, rtM204V, rtA181T 30%/V 20%/wt 50% and rt T184G 40%/wt 60%, were identified by sequence analysis (Applied Biosystems 3500 Series Genetic Analyzer and Pyrosequencing PSQ 96MA;Life Technologies). Combination treatment with entecavir 0.5mg plus tenofovir 300mg daily was immediately selected in month 133. Viral load and serum creatinine gradually decreased, and CC correspondingly increased. Four months later, treatment with entecavir plus tenofovir resulted in undetectable HBV DNA with <20IU/ml, normal ALT and AST, and CC 76.5ml/min (Figures 1 and 2).

## Discussion

Ideal NA therapy in CHB patients with kidney transplantation must ensure virological suppression with undetectable HBV DNA and minimize renal injury, which will prevent the progression of liver disease and maintain anti-rejection therapy after kidney transplantation. However, resistance to NAs frequently results in virological breakthrough, acute exacerbation of liver disease, and complicated deterioration of the transplanted kidney. The emergence of drug resistance and the subsequent need for rescue therapy increases the risk of developing HBV multidrug resistance. In this report, a CHB patient with kidney transplantation received sequential monotherapy with NAs and inappropriate combination therapies, which caused multidrug resistance and renal functional injury. An alternative combination therapy with entecavir plus adefovir was administered in month 106. As a result of rebounded HBV load and significantly declining CC, combination therapy with tenofovir plus entecavir was administered in month 133. After eight weeks, undetectable HBV DNA, normal hepatic function, and improvement of CC were present. Compared with combination therapy with adefovir plus entecavir, tenofovir plus entecavir shows a potent antiviral effect for multidrug resistance and minimized renal injury in patients with kidney transplantation.

All NAs are cleared by the kidneys and dose adjustments are recommended according to the patient’s CC, as listed in the drug information. Because tenofovir had not yet been approved in China by 2011, we alternatively chose combination therapy with entecavir plus adefovir in month 106, in an attempt to inhibit HBV replication and maintain stable CC. If CC was <50ml/min, adefovir dosage would be simultaneously regulated according to the drug information for adefovir. However, our patient’s CC was >50ml/min during the entire period of therapy, therefore adefovir dosage was not adjusted. Combination therapy with entecavir 1mg plus adefovir resulted in low viral load and generally normal CC during 28 months. Unfortunately, this combination therapy did not completely inhibit HBV replication, and eventually induced viral rebound and renal function decline.

Entecavir and tenofovir, as potent HBV inhibitors with a high barrier to resistance, are recommended as first-line monotherapy for CHB patients [1,2]. However, entecavir has low efficacy in cases of lamivudine or telbivudine resistance, and thus tenofovir may be a better option in patients with lamivudine resistance, including those with kidney transplantation [5]. For patients with multidrug resistance, tenofovir plus entecavir may be preferred [2]. Thus, tenofovir plus entecavir was administered to inhibit multidrug resistance in the present case, and induced a rapid decrease in HBV DNA and HBeAg and improvement of renal function during four months (Figures 1 and 2).

Currently, tenofovir is used to treat CHB patients with kidney transplantation or chronic kidney disease, such as in the investigation of Daudé and case report of Das in 2011 [6,7]. Tenofovir treatment showed excellent efficacy in inhibiting HBV and in maintaining renal function. Recently, Pipili has suggested that tenofovir is a better option in kidney transplant patients with drug resistance [5]. In our report, tenofovir treatment of a CHB patient with kidney transplantation was also safe and efficacious, consistent with the previous results. In addition, the case report of Das showed that a patient with nephrotic syndrome and HBV infection was treated by tenofovir plus lamivudine, resulting in arrest of proteinuria and stabilization of renal function [7]. The data of Das were similar to our present report, in that tenofovir inhibited HBV and improved renal function, simultaneously.

Side effects of tenofovir are mainly proximal tubular injury namely Fanconi’s syndrome, isolated hypophosphatemia, and decreased bone mineral density [8]. Some authors have reported that 15% of patients with tenofovir treatment lasting two to nine years developed renal tubular dysfunction associated with acute or chronic kidney injury, but this illness might be reversible [9-11]. In a multicenter, prospective cohort study, only small nonprogressive decreases in renal function were observed in 3% of patients with tenofovir treatment lasting five years [12]. Therefore, renal function, electrolytes, and bone mineral density should be periodically detected in long-term therapy with tenofovir.

In addition, we discussed the inadequate or mistaken NAs therapies in this case, including: (1) adefovir monotherapy followed by lamivudine resistance (2) sequential monotherapy with lamivudine, adefovir, telbivudine and entecavir and (3) combination therapy with lamivudine plus entecavir or pegylated IFN plus entecavir. Currently, many authors have suggested that sequential monotherapy with antiviral agents with low barriers and hence high or intermediate risk of resistance (lamivudine, adefovir and telbivudine) should be strictly avoided, because of the increasing risk of multidrug resistance [2]. In patients with monodrug resistance, appropriate rescue therapy should add an effective antiviral agent that does not share cross-resistance to combination therapy. For lamivudine resistance, combination therapy with lamivudine plus adefovir is an option [2,4]. However, the rescue therapy for lamivudine resistance in the present case in month 51 was switching to adefovir, instead of adding it. Subsequently, a series of sequential monotherapies and mistaken combination therapies caused multidrug resistance. As a result of the shared resistance mutations between telbivudine and lamivudine, monotherapy with telbivudine in months 60 to 68 was mistaken. The incorrect combination therapy with lamivudine and entecavir in months 87 to 102 also contributed to the multidrug resistance. Even some infectious disease physicians have proposed combination therapy with pegylated IFN plus entecavir to treat multidrug resistance in month 105. This mistaken therapeutic strategy has doubtless been rejected by renal physicians. Two factors have led to the above regimens. First, physicians in various hospitals, including those for infectious disease and renal disease, and our own hospital, have separately participated in NA therapy in different phases, but not all physicians understand the basic principles of resistance, pharmacokinetics of NAs, and management strategies for kidney transplantation. Therefore, no systematic rescue therapy for resistance mutation was determined during the 55 months. Second, tenofovir had not been used to treat HBV resistance before 2012 in China. That is why we alternatively chose combination therapy with entecavir 1mg plus adefovir 10mg daily to treat multidrug resistance in month 106.

The mechanism of sequential monotherapy inducing multidrug resistance has partially been found in recent years. Coffin and colleagues have detected HBV replication, drug resistance mutations, and gene diversity in three different tissues of liver transplant patients with NA therapy, including explanted liver, peripheral blood mononuclear cells (PBMCs), and plasma. Their data showed 100% wild type in plasma, 83% wild type in PBMCs, but 66% drug resistance in the liver, which was defined as compartmentalization of HBV variants. Notably, three patients who experienced lamivudine or adefovir and complicating resistance mutation were administered adefovir or tenofovir for rescue therapy lasting six to 19 months. The rescue therapy of adefovir or tenofovir resulted in undetectable HBV DNA and 100% wild type in plasma, but retained detectable HBV DNA, covalently closed circular DNA (cccDNA) and resistant mutations correlating with lamivudine or adefovir in explant liver. Despite apparent HBV suppression in plasma, the liver continued to support viral replication and harbor resistant viruses. On sequence and phylogenetic analysis of HBV P gene in the liver, the authors further demonstrated that drug resistance correlated with increased HBV quasispecies diversity [13]. In the study of Liu *et al.*, the authors analyzed the quasispecies complexity of HBV in plasma of patients with entecavir treatment, in which all patients were divided into responders and partial responders. After four weeks, quasispecies complexity in the responders decreased, while in partial responders it increased. High quasispecies complexity was correlated with increased cumulative probability of drug resistance [14]. Consistent with the studies of Coffin *et al.* and Liu *et al.*, initial monotherapy with lamivudine in the present case induced increased quasispecies complexity, lamivudine genetic resistance, and virological breakthrough. The cccDNA of lamivudine-resistant viruses might play a dual role, as the template for transcription of pregenomic RNA, and persistence of resistance mutation in the nucleus of hepatocytes. Subsequently sequential monotherapy caused accumulation of resistance mutation by repeated replication cycle, and eventually resulted in multidrug resistance. During NA therapy, hepatocytes harbor multidrug-resistant viruses.

## Conclusions

In CHB patients with kidney transplantation or chronic kidney disease sequential monotherapies with antiviral agents with low barriers should be avoided, and initial therapy with entecavir is a better option. Compared with combination therapy with adefovir plus entecavir, tenofovir plus entecavir for kidney transplant patients with multidrug resistance is safe and effective.

## Consent

Written informed consent was obtained from the patient for publication of this case report and any accompanying images. A copy of the written consent is available for review by the Editor-in-Chief of this journal.

## Abbreviations

ADV: adefovir; ALT: alanine aminotransferase; AST: aspartate aminotransferase; CC: creatinine clearance; cccDNA: covalently closed circular DNA; CHB: chronic hepatitis B; ETV: entecavir; HBV: hepatitis B virus; IFN: interferon; HBeAg: hepatitis B e antigen; LdT: telbivudine; LVD: lamivudine; NAs: nucleos(t)ide analogs; PBMC: peripheral blood mononuclear cell; TDF: tenofovir; ULN: upper limit of normal; wt: wild type.

## Competing interests

The authors declare that they have no competing interests.

## Authors’ contributions

CS carried out the clinical treatment, follow-up and collection of data, data analysis and manuscript writing. GQY and PW were responsible for the conception and design, and final approval of the manuscript. All authors read and approved the final manuscript.
